# A male fetus with cyclopia was discovered after miscarriage: A rare case report from Syria

**DOI:** 10.1002/ccr3.8644

**Published:** 2024-03-10

**Authors:** Tala Dakkak, Marah Mansour, Habib Jarbouh, Abdolmoin Ktail

**Affiliations:** ^1^ Faculty of Medicine Hama University Hama Syrian Arab Republic; ^2^ Faculty of Medicine Tartous University Tartous Syrian Arab Republic; ^3^ Division of Colon and Rectal Surgery, Department of Surgery Rochester Minnesota United States of America; ^4^ Faculty of Medicine Damascus University Damascus Syrian Arab Republic; ^5^ Department of Pathology Maternity University Hospital Damascus Syrian Arab Republic; ^6^ Department of Obstetrics and Gynecology, Alasaad Medical Complex Hama Syrian Arab Republic

**Keywords:** alobar holoprosencephaly, case report, cyclopia, malformation, synophthalmia

## Abstract

**Key Clinical Message:**

This case of alobar holoprosencephaly and cyclopia emphasizes the value of prenatal check‐ups, particularly in low‐income countries. Early ultrasound diagnosis leads to early gestational termination, preventing psychological trauma for the parents.

**Abstract:**

Alobar holoprosencephaly is a rare‐occurrence malformation with a bad prognosis linked to cyclopia, the most severe cranial feature. Prenatal examinations are essential for identifying these deformities and preventing parental mental health damage.

## 1 INTRODUCTION

Cyclopia (synophthalmia) is an uncommon fatal congenital abnormality.^1^, ^2^, ^3^ It is the most severe case of holoprosencephaly (HPE).^1^, ^3^, ^4^, ^5^, ^6^, ^7^ During the organogenesis in antenatal life, the embryonic prosencephalon is partially divided or fails to divide into the right and left cerebral hemispheres,^2^, ^3^ which leads to the failure of orbits to divide into double separate caves.^1^, ^4^, ^7^, ^8^, ^9^ This happens between the eighteenth and the twenty‐eighth day of gestation.^1^, ^4^, ^7^, ^8^ The incidence is 1.05 per 100,000 births, including stillbirth^1^ with a tendency to occur in females.^8^ Clinically, there are typical craniofacial features, including a single or imperfectly cleft eye in one orbit, nasal agenesis or proboscis, which is a nonfunctional nose located above the central orbit and that is known as Rhinocephaly.^1^, ^2^, ^3^, ^6^, ^9^, ^10^ Furthermore, extra‐cranial characteristics are also presented along with Cyclopia such as polydactyly, renal dysplasia, Omphalacele^1^, ^2^, ^6^ and cardiac defects such as ventricle septum defect (VSD).^10^ Synophthalmia is possible to develop for no specific reason^7^ or due to risk factors, including exposure to deformed substances during pregnancy,^10^ diabetes, infections,^6^ and genetic mutations.^6^, ^10^ Patau syndrome (trisomy 13) is the most common chromosomal syndrome associated with cyclopia.^3^, ^10^ This case is incompatible with life^4^, ^5^, ^7^ and even newborns die within several hours after birth.^1^, ^3^, ^8^, ^10^ Antenatal diagnosis begins from the 22nd week of pregnancy^6^ by ultrasound (US).^4^, ^5^, ^6^, ^7^ Herein, we present a unique case of alobar HPE with cyclopia which was diagnosed after miscarriage. To the best of our knowledge, this is the first documented case from Syria.

## 2 CASE HISTORY

A 29‐year‐old female G1P1 presented to the Obstetrics and Gynecology Department with a sudden absence of in‐pregnancy symptoms such as pregnancy craving, vomiting, and nausea, accompanied by lower abdominal pain and mild spotting. There were no other symptoms. The mother had a partus caesarius 2 years ago. The mother had smoked three packs per year for 6 years. There was no other medical, family, surgical, or allergic history. The mother was at 12^+2^ weeks gestation based on her last menstrual cycle and had never had an US before during this pregnancy.

## 3 METHODS

The US demonstrated a non‐viable intrauterine fetus at age 11 weeks gestation, with irregularities in the skull bones and severe cerebral malformation. A single orbital cave was also noted. The US investigation was ineffective. 800 mg of Misoprostol was prescribed to induce medical miscarriage (400 mg oral and 400 mg vaginal).

## 4 CONCLUSION AND RESULTS

**FIGURE 1:**
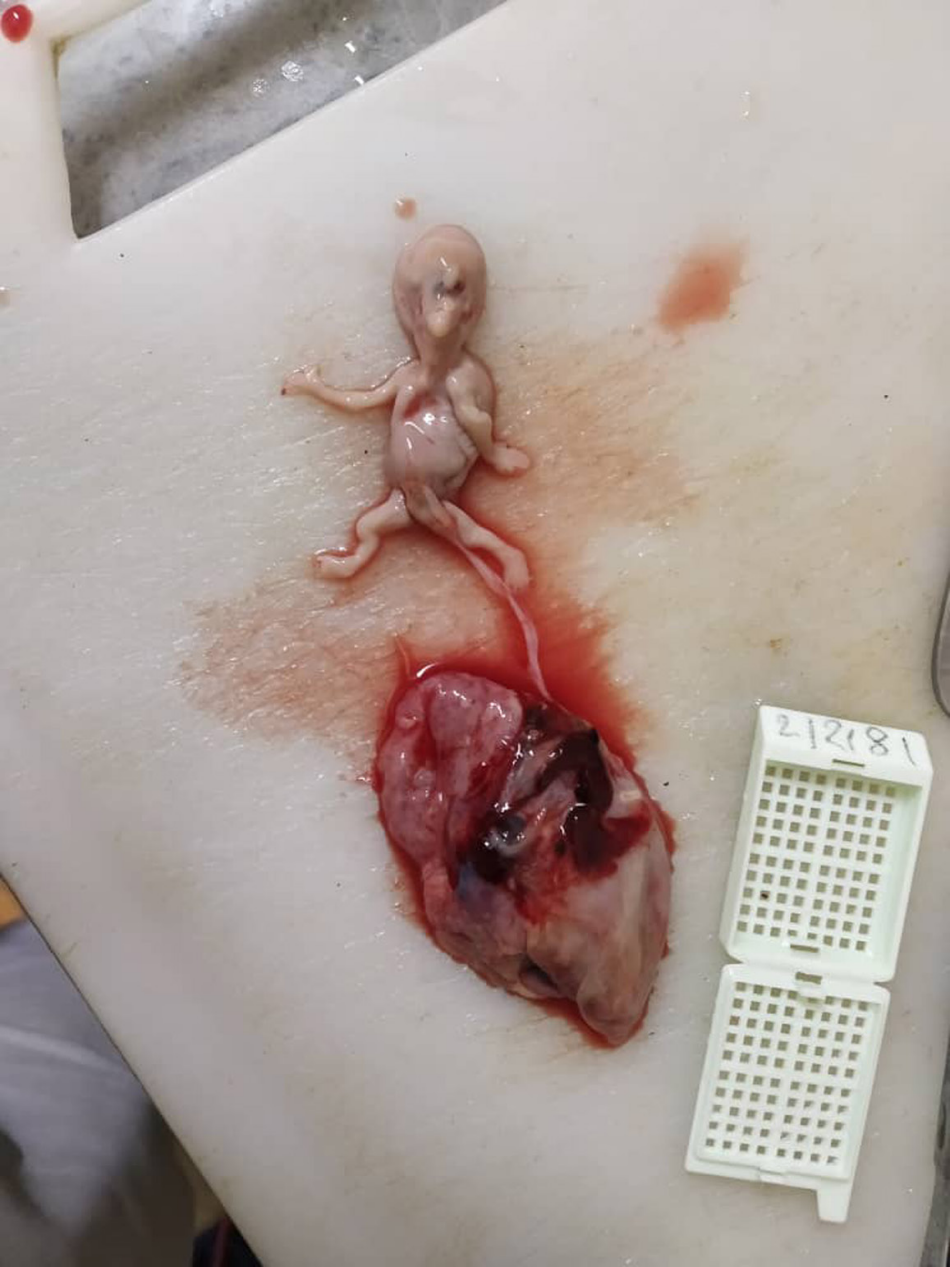
Anomalous abortion with normal placenta.

**FIGURE 2:**
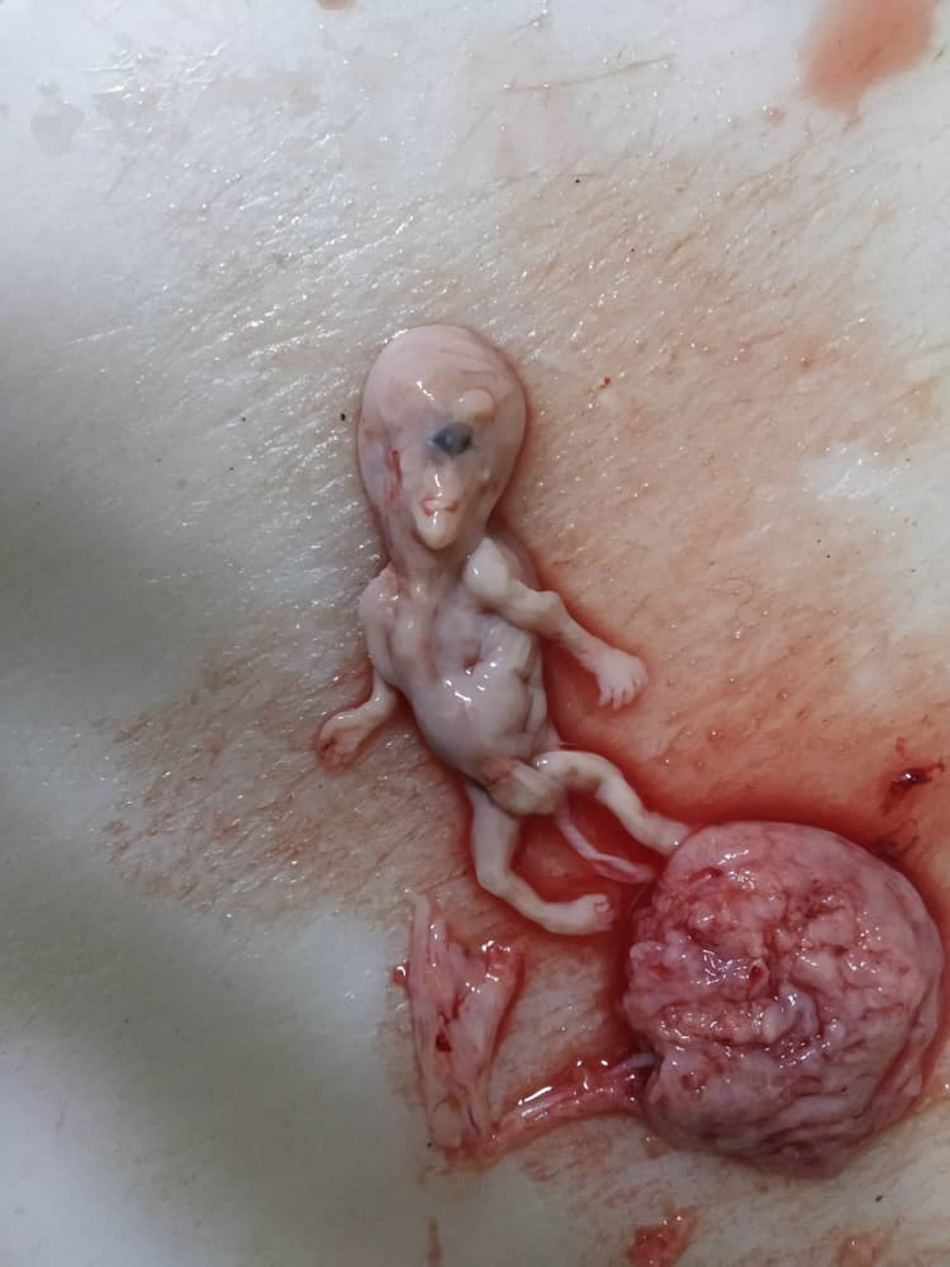
A single eye with proboscis above the eye.

**FIGURE 3:**
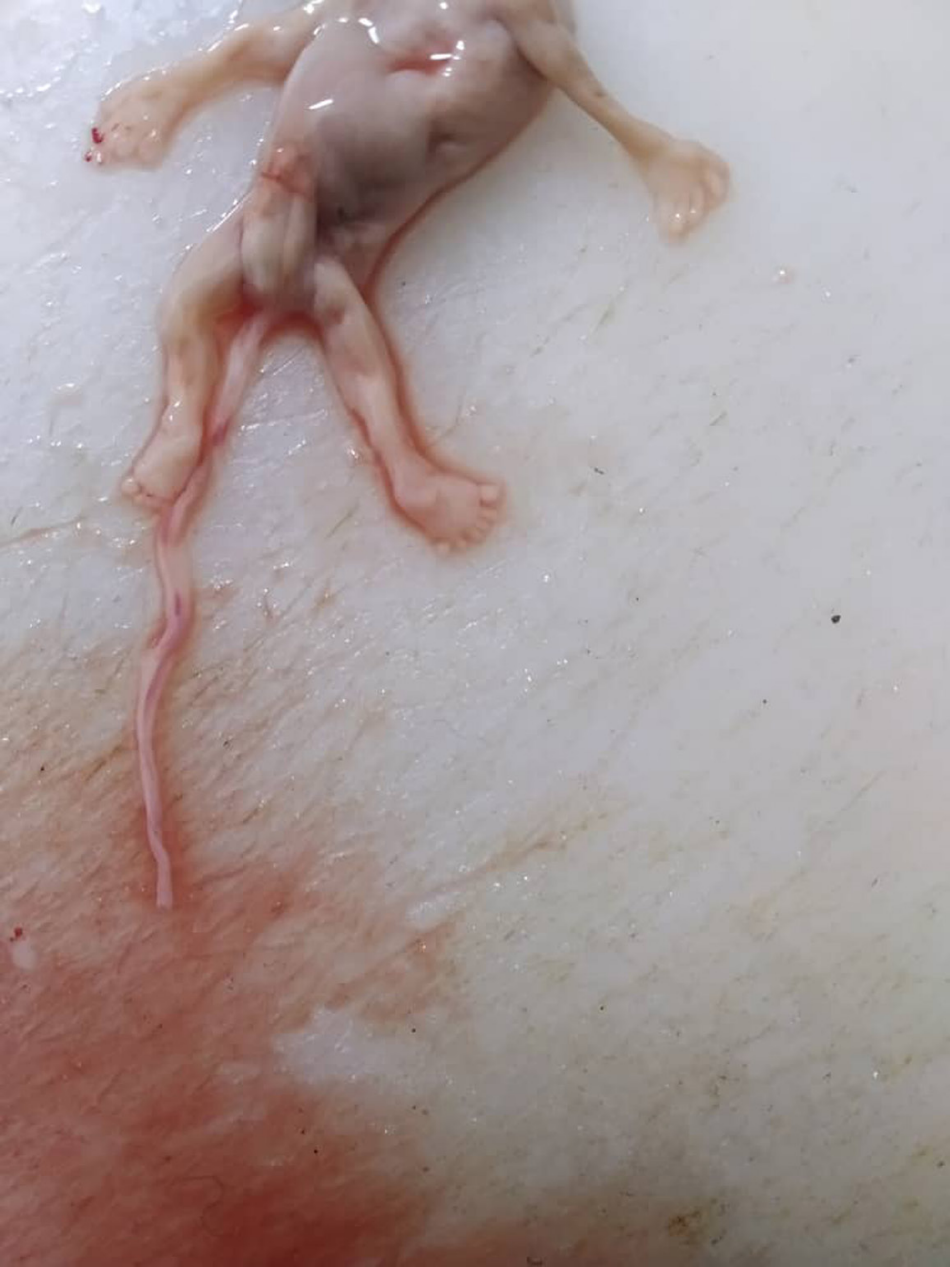
A postaxial polydactyly in the left foot.

The pathological exam demonstrated a male embryo measuring 7 cm in length (Figure 1). Gross examination showed a single central orbit located in the middle of the face with a cutaneous horn above it (Figure 2); and polydactyly in its left foot, attached with a normal umbilical cord (Figure 3). The visceral organs of the embryo were normal except the brain's front, which showed adhesion of its right and left hemispheres. A placenta measures 7 × 2 cm and is composed of normal villi and decidual tissue (Figure 1).

## 5 DISCUSSION

HPE is a rare complex congenital malformation that occurs in the human brain during organogenesis of embryonic development in which the primitive brain has a role in the development of the orbits, completely or partially fails in division into right and left hemispheres,^3^, ^4^, ^5^, ^6^, ^8^, ^10^ and that typically happens between the 18th and 28th day of gestation,^1^, ^6^, ^7^ which causes a group of craniofacial anomalies.^5^ There are three types of HPE depending on the severity of the case: Lobar, characterized by the separation of the left and right ventricles with a degree of frontal cortical continuity, semi‐lobar where there is an incomplete separation, and alobar where the interhemispheric fissure is absent there is only one cerebral ventricle.^10^ The alobar HPE is considered the most severe form due to its manifestation which also includes: undifferentiated cerebral hemispheres, a thalamic fusion,^2^, ^4^ a missing corpus callosum, absence of olfactory nerves or optic tracts,^4^ and cyclopia,^8^ which is the rarest and most severe facial expression of alobar HPE.^1^, ^3^, ^7^ Cyclopia is defined by the fusion of two optic grooves^1^ as a result of the embryonic prosencephalon's improper division of the eye's orbits into two cavities.^3^, ^6^, ^7^ The term “cyclopia”, originally from Greek mythology, refers to anarchist giant shepherds that had a single round eye on their front.^8^ Cyclopia itself is always an outward sign of a profound brain abnormality.^4^ Due to the embryonic forebrain and mid‐face both deriving from the prechordal mesoderm, several facial malformations are typically linked, along with several other defects.^10^ The prevalence of newborns with this condition, including stillbirths is 1.05 per 100,000 live births^1^ and there is a preponderance of female babies.^7^, ^8^, ^11^ In our case, it was a male stillborn. Nevertheless, according to recent studies, there is a balanced sex ratio among fetuses identified prenatally during the gestational period ranging from 16 to 36 weeks suggesting that there is no significant disparity in the rate of fetal loss between males and females at this advanced stage of pregnancy.^12^ The etiology of this condition is still unknown exactly.^1^, ^2^, ^7^, ^9^ Multiple heterogeneous risk factors are related. Both genetic and environmental factors are possible including teratogenic medication exposure during pregnancy (anticonvulsants, aspirin, retinoic acid, aspirin, lithium), maternal diabetes, alcohol consumption, infections including toxoplasmosis, rubella, cytomegalovirus, and herpes simplex (TORCH), and chromosomal defects.^1^, ^2^, ^6^, ^7^, ^8^, ^9^ As the fetal neurological system's development depends on thyroid hormone, therefore the connection may make biological logic between thyroid hormone deficiency and HPE.^12^ The dynamic and tissue‐specific activation of the Sonic Hedgehog (SHH) pathway, as well as other signaling pathways including Fibroblast Growth Factor (FGF), Bone Morphogenetic Protein (BMP), and Wingless‐Type MMTV Integration Site (WNT) families, plays a crucial role in orchestrating tissue morphogenesis during early stages of facial development. SHH signaling plays a crucial role in regulating tissue development and patterning, as well as in maintaining cell viability. Previous studies have identified several key downstream mediators of SHH signaling that are involved in different stages of craniofacial development. These findings highlight the intricate and essential role of SHH signaling in orchestrating the complex processes underlying craniofacial morphogenesis.^13^ Despite the limitation of evidence, the SHH gene regulator was revealed to be involved in the division of the single eye field into two bilateral fields, so when mutations cause SHH muting, the result will be cyclopia with the eye fused in the middle of the face.^3^, ^6^, ^8^ Prior research has elucidated that the simultaneous deletion of multiple Hedgehog co‐receptors in certain tissues leads to a more pronounced phenotype compared to the inactivation of a single gene. To enhance our comprehension of the precise regulation of SHH signaling activity, further comprehensive and tissue‐specific investigations into SHH's co‐receptors and modifiers are warranted. These investigations have the potential to deepen our understanding of the complex regulatory mechanisms governing SHH signaling and its impact on tissue morphogenesis during facial development.^13^

Another potential risk factor for adverse pregnancy outcomes is the residence of parents in radiation‐prone areas and their occupation in the mining industry. Exposure to ionizing radiation, a known teratogen, in such environments can increase the risk of genetic mutations and developmental abnormalities in the fetus. Studies have shown a correlation between maternal exposure to radiation and an increased incidence of congenital anomalies.^11^ However, in this presented case, no risk factors could be detected. Alobar HPE is associated with many syndromes such as Smith‐Lemli‐Opitz Syndrome (SLOS), Pallister‐Hall Syndrome, and Trisomy 13 ‘Patau Syndrome’,^2^, ^3^, ^10^ which is the most common cause of HPE.^10^ The facial deformities associated with alobar HPE may include cyclopia, a single orbit with a median single eye, or a partly split eye.^6^, ^10^ The nose is either absent completely or replaced with a proboscis as a non‐functioning nose.^1^, ^3^, ^7^, ^10^ Typically, such a proboscis is observed on the back or above the central eye and is a defining feature of a form of cyclopia known as rhinocephaly or rhinencephaly.^1^ Missing philtrum, otocephaly, astomia, or microstomia also could be found.^6^ In our case, we found the typical facial features of cyclopia that included: a median single orbit, the absence of a nose, and a proboscis above the eye (Figure 2). The presence of extra‐facial deformities like polydactyly, renal dysplasia, omphalocele,^6^, ^7^, ^8^ ventricular septal defects, and myelomeningocele is reported in other literature.^10^ Only polydactyly in the left foot could be observed in our dead fetus (Figure 3). Karyotyping and other genetic tests are commonly employed in high‐income nations for the evaluation of the severity of syndromic HPE. These diagnostic tools play a crucial role in providing valuable information for genetic counseling and facilitating preventive measures to mitigate the risk of recurrent occurrences within affected families.^11^ During the first trimester, the US can reveal distinctive images that makes it the most helpful investigation for diagnosing cyclopia.^1^, ^2^, ^4^, ^6^, ^7^, ^9^ After the third or fourth week of pregnancy, the US can typically detect clear indications of cyclopia or other forms of HPE.^1^ In most cases that were reported, the anomaly was detected early during the anomaly scan.^2^ When HPE is suspected by the US, careful intrauterine scanning of the fetus's face can lead to a more accurate diagnosis.^4^ However, the usual US may not detect certain features that are useful in diagnosis,^7^ thus obtaining more information about the development of brain structures is possible through in‐utero magnetic resonance imaging (MRI)^4^ or high‐resolution MRI scans,^7^ which is considered the gold standard due to its exceptional resolution in visualizing soft tissue structures.^11^ However, in our case, the mother had never had any prenatal care, and the anomaly was only discovered after the fetus was miscarried. The survival rate is extremely low in this condition^10^ and the prognosis depends on brain fusion degree, malformation, and complications.^2^, ^4^ In cases of lobar HPE, children can survive for several years with neurological and mental challenges. Both alobar and semi‐lobar HPE have been associated with the worst prognosis^2^ and are not compatible with life.^2^, ^4^, ^7^ Generally, the result will either be a miscarriage or a stillborn, and even if babies are born alive, they only survive a few hours after birth.^2^, ^3^, ^6^, ^7^ The occurrence of pregnancy loss complicates the identification of non‐genetic risk factors.^12^ The fetus, in our case, presented with the alobar form of HPE associated with cyclopia (Figure 1), Without being genetically tested after the medical expulsion. In all cases, termination of the pregnancy should always be offered as an option for management. This procedure follows a comprehensive prenatal examination and relevant genetic counseling, owing to the severity of the defects. Further aids in the diagnosis of cyclopia include postnatal chromosomal analysis and gross examination of the specimen.^6^, ^10^ There is no currently known treatment for this condition, and there is no way to prevent it.^8^ This report emphasizes the importance of prenatal check‐ups, particularly in developing countries, and the significance of early US diagnosis for gestation termination and maternal psychological trauma prevention. In conclusion, Cyclopia is a rare lethal abnormality that occurs in the early stages of pregnancy, and this condition is incompatible with life. Routine check‐ups during gestation have an important value in helping identify fetuses with anomalies and lead to pregnancy termination after the parent's approval.

## AUTHOR CONTRIBUTIONS

**Tala Dakkak:** Conceptualization; data curation; formal analysis; investigation; methodology; project administration; resources; software; validation; visualization; writing – original draft; writing – review and editing. **Marah Mansour:** Conceptualization; data curation; formal analysis; investigation; resources; software; writing – original draft; writing – review and editing. **Habib Jarbouh:** Project administration; resources; validation; writing – original draft; writing – review and editing. **Abdolmoin Ktail:** Project administration; resources; supervision; validation; writing – original draft; writing – review and editing.

## FUNDING INFORMATION

The authors declare no source of funding for this manuscript from any organization or institution.

## CONFLICT OF INTEREST STATEMENT

The authors declare that there is no conflict of interest and none of them is or was employed by any government agency that has any function other than research and education, and none of them is submitting this manuscript as an official representative or on behalf of the government.

## ETHICS STATEMENT

Not applicable to this case report.

## CONSENT

Written informed consent was obtained from the patient for publication of this case report and accompanying images. A copy of the written consent is available for review by the Editor‐in‐Chief of this journal on request.

## Data Availability

All data on which the conclusions of this case report are based are included in this manuscript.
